# Idebenone and Resveratrol Extend Lifespan and Improve Motor Function of HtrA2 Knockout Mice

**DOI:** 10.1371/journal.pone.0028855

**Published:** 2011-12-19

**Authors:** Ellen Gerhardt, Simone Gräber, Éva M. Szegő, Nicoleta Moisoi, L. Miguel Martins, Tiago F. Outeiro, Pawel Kermer

**Affiliations:** 1 Department of NeuroDegeneration and Restorative Research, DFG Research Center: Molecular Physiology of the Brain, University Medicine Göttingen, Georg-August University, Göttingen, Germany; 2 High Throughput Screening Unit, Leibniz-Institut für Molekulare Pharmakologie, Berlin, Germany; 3 Cell Death Regulation Laboratory, Toxicology Unit , Medical Research Council, Leicester, United Kingdom; 4 Department of Neurology, DFG Research Center: Molecular Physiology of the Brain, University Medicine Göttingen, Göttingen, Germany; University of Windsor, Canada

## Abstract

Heterozygous loss-of-function mutation of the human gene for the mitochondrial protease HtrA2 has been associated with increased risk to develop mitochondrial dysfunction, a process known to contribute to neurodegenerative disorders such as Huntington's disease (HD) and Parkinson's disease (PD). Knockout of HtrA2 in mice also leads to mitochondrial dysfunction and to phenotypes that resemble those found in neurodegenerative disorders and, ultimately, lead to death of animals around postnatal day 30. Here, we show that Idebenone, a synthetic antioxidant of the coenzyme Q family, and Resveratrol, a bioactive compound extracted from grapes, are both able to ameliorate this phenotype. Feeding HtrA2 knockout mice with either compound extends lifespan and delays worsening of the motor phenotype. Experiments conducted in cell culture and on brain tissue of mice revealed that each compound has a different mechanism of action. While Idebenone acts by downregulating the integrated stress response, Resveratrol acts by attenuating apoptosis at the level of Bax. These activities can account for the delay in neuronal degeneration in the striata of these mice and illustrate the potential of these compounds as effective therapeutic approaches against neurodegenerative disorders such as HD or PD.

## Introduction

Mitochondrial dysfunction has been implicated in the pathogenesis of several neurodegenerative disorders such as Huntington's disease (HD) and Parkinson's disease (PD). These disorders are characterized by the selective loss of neurons, accumulation of reactive oxygen species (ROS), loss of mitochondrial membrane potential, and ATP depletion [1], [2].

Administration of environmental toxins, like 1-methyl-4-phenyl-1,2,3,6-tetrahydropyridine or rotenone, *in vivo* is able to mimic some pathological features of neurodegenerative disorders, suggesting a cross-talk between mitochondrial dysfunction and the ubiquitin proteasome system (UPS) [3].

HtrA2 (Omi) is a serine/threonine protease residing in the intermembrane space of mitochondria attached to the inner membrane. The absence of HtrA2 causes a parkinsonian phenotype in knockout mice, which is characterized by an accumulation of unfolded proteins inside mitochondria and [4] defective mitochondrial respiration and enhanced ROS, either of which can induce neuronal death through activation of the internal stress response (ISR) by upregulation of the transcription factor CHOP [5], [6]. Notwithstanding, the discussion remains on whether HtrA2 is a susceptibility gene for neurodegenerative disorders, although animal studies provide strong evidence for a neuroprotective function for HtrA2 [7], [8], [5].

In line with this view, HtrA2 knockout (KO) mice exhibit pronounced akinesia-like movement-related symptoms starting on day 23 after birth. They show a focal loss of neurons with reactive astrogliosis in the striatum and a selective loss of terminals in the nigrostriatal pathway. At the time of death, which occurs on average on post-natal day 28, neuronal degeneration is most pronounced in the striatum but seems to progress, albeit at a slower pace, in many areas of the brain including the forebrain and (possibly) cerebellum [5].

To further validate the defect in oxidative stress resistance in HtrA2 KO mice, we studied the effects of two compounds (Idebenone and Resveratrol) on the disease course and on the lifespan of HtrA2 KO animals and correlated these results with expression levels of CHOP in different cell death models of oxidative stress. In this regard, Idebenone is thought to have antioxidant properties similar to CoQ10 which is in use for anti-aging products on the basis of the free-radical theory [9]. Resveratrol (3,5,4′-trihydroxystilbene) is both an antioxidant and anti-inflammatory substance [10]. Interestingly, both Idebenone and Resveratrol ameliorate disease symptoms in HtrA2 KO mice while they show different effects on CHOP expression levels, further emphasizing the need for studies dissecting the various pathways mediating pathology in neuronal disorders.

## Materials and Methods

### Ethics statement

All protocols are in accordance with the animal research protocol 33.11.42502-04-117/08 of Niedersachsen and were constantly supervised by veterinarians of the University Medical Center, Goettingen.

### Oral administration of Idebenone and Resveratrol

Since both drugs are known to pass the blood brain barrier after oral administration, they were administered by mixing the drugs with the regular feed of the animals [4], [11], [12], [13].

A powder of feeding pellets was mixed with sugar and water. A small portion of this sweet mash was prepared for each cage. The animals prefer the sweetened food over the regular food and usually completely ingested the portion of mash within minutes of placing it into the cage. Idebenone or Resveratrol were suspended in 0.5% methylcellulose (Sigma, Taufkirchern, Germany) and mixed into the mash, at 500 mg/kg body weight for Idebenone and as 25 mg/kg body weight for Resveratrol per day. Concentrations of Idebenone and Resveratrol were chosen in accordance to previous reports [14], [11]. Untreated controls received the same amount of sweet mash without drugs. From birth till weaning a single mother and her pups were housed in one cage. Only the mother ate the mash, so the amount of drug was adjusted to the body weight of the mother to yield the final concentration. Both drugs can pass placenta, but the drug concentration that pups received during this time was not determined.

On post-natal day 5, pups received paw tattoos for identification and KO mice were identified via PCR [6].

From post-natal day 20, mice were housed singly, and received the sweet mash with or without drug adjusted for their body weight. Animals were sacrificed when they were unable to walk. Often, spontaneous death occurred before paralysis. In both cases, day of death were noted. Mice used for longevity and motor task experiments were sacrificed by carbon dioxide asphyxia.

For statistical analysis one-way ANOVA was carried out followed by Mantel-Cox Test.

### Motor task

Mice were placed into a modified inverted grid apparatus [15]. Mice were placed into the box with grid floor in the upright position. Then the box was inverted and the time the mice could cling to the grid floor was measured. The experiment was conducted every other day, starting one day after weaning. Each day consisted of 4 trials each, and the longest time the mouse could cling to the grid was recorded. Maximum time per trial was one minute. All mice, even untreated KO mice, were able to complete at least one trial of one minute shortly after weaning. Successful completion of the motor task was defined as the ability to hang onto the grid for 10 sec or more.

### Immunohistochemistry and stereology

For immunohistochemistry and molecular biological analysis, six mice/treatment group were sacrificed using CO_2_. Brains were rapidly removed, and dorsal striata from both hemispheres were dissected on ice. One hemisphere of each brain was used for mRNA analysis and the other hemisphere was fixed in 4% paraformaldehyde for 48 hrs. After cryoprotection (30% sucrose), 30 µm coronal slices were cut in cryostat. For quantification of neuron number in the striatum, free-floating, peroxidase based immunohistochemistry was performed as we described previously [16]. Briefly, every fifth sections was treated with anti-neuronal nuclei (anti-NeuN) antibody (1∶1000, Millipore, Billerica, MA, USA) for 48 h at 4°C, then treated with biotinylated anti-mouse IgG (1∶200; Vector Laboratories, Burlingame, CA, USA) followed by avidin–biotin–horseradish peroxidase (HRP) complex (1∶500, Vector Standard Elite Kit, Vector Laboratories, Burlingame, CA, USA). Peroxidase labeling was visualized by diaminobenzidine tetrahydrochloride. The omission of primary or secondary antibodies resulted in a complete absence of immunoreactivity (ir).

The total number of NeuN-positive neurons in the dorsal striatum [17] was counted (plate 21–31) by using the optical fractionator, an unbiased stereological technique of cell counting (StereoInvestigator, MBF Bioscience, Madgeburg) [18], [19]. Counts were performed blinded for experimental grouping, by using an oil immersion 63× objective (Axioskop 2 microscope, Zeiss, Jena, Germany), a counting frame of 50× 50 µm and a grid size of 100×100 µm. Six slices/animal were used in the analysis, and the size of the striatum was identical in wild-type and KO animals.

The Two-way ANOVA followed by Newman–Keuls *post hoc* test were carried out.

### Realtime PCR analysis

cDNA was reverse transcribed from RNA extracted from cells (cerebellar granule neurons) or from striatum derived from wt or HtrA2 KO mice [5]. Real time PCR was performed by using Mesa Blue qPCR MasterMix Plus for SYPR Assay low rox (Eurogentec, Belgium). Primers for amplification of several genes were designed by using the software Primer3 and are listed in Table 1.

**Table 1:** Primers used in this study.

Gene	forward	reverse
**CHOP**	5′-CGGCCTGGGAAGCAACGCAT-3′	5′-GTCGATCAGAGCCCGCCGTG-3′
**ATF4**	5′-GGGTTCTGTCTTCCACTCCA-3′	5′-GGGCTCATACAGATGCCACT-3′
**p53**	5′-CACAGCGTGGTGGTACCTTA-3′	5′-TCTTCTGTACGGCGGTCTCT-3′
**Noxa**	5′-CGCGCAGAGCTACCACCTGA-3′	5-TCCGGAGTTGAGCACACTCGT-3′
**Bcl_2_**	5′-TCGCAGAGATGTCCAGTCAG-3′	5′- ATGCCGGTTCAGGTACTCAG-3′
**Bax**	5′-ATGGAGCTGCAGAGGATCAT-3′	5′- GATCAGCTCGGGCACTTTAG-3′
**DR5**	5′GTCAGAAGGGAACTGCAAGC-3′	5′ -TGCATCGACACACCGTATTT-3′
**β-actin**	**5′**-GCGAGAAGATGACCCAGATC-3	**5′**- CCAGTGGTACGGCCAGAGG-3

### Statistical analysis

Data were analyzed using GraphPad Prism 4.0 (GraphPad Software Inc.), using one-way ANOVA followed by Bonferroni's Multiple Comparison Test (real-time analysis), when significant (p<0.05), unless otherwise noted. Statistical significance referred to as: * p<0.05; ** p<0.01; *** p<0.001. All data are presented as mean ± SD.

**Figure 1:**
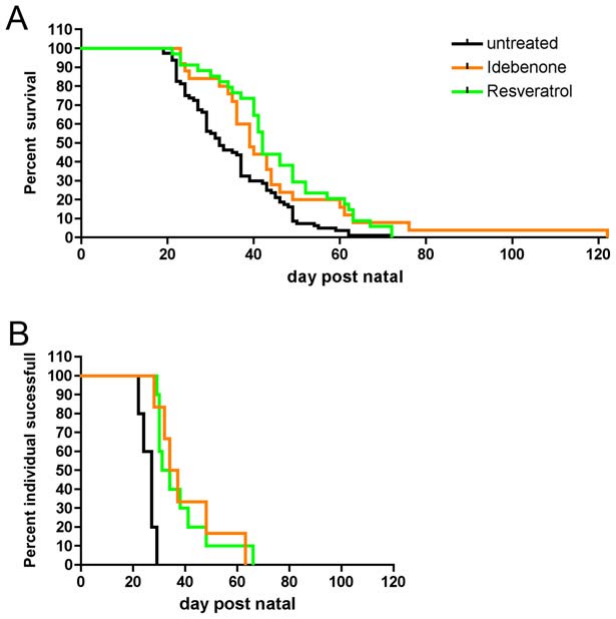
Treatment of HtrA2 KO animals with Idebenone and Reseveratrol. **A** Both Idebenone (orange line, 25 animals) and Reseveratrol (green line, 34 animals) significantly increased the mean survival time of HtrA2 KO mice compared to sham-treated animals (black line, 77 animals,). In addition, Idebenone increased the maximal life span. **B** Grid test of HtrA2 KO animals. Both compounds extend the period of life during which the KO animals can perform the motor task successfully (Idebenone: 10 animals, resveratrol: 6 animals, untreated 5 animals).

For comparison of the curves in Fig.1a and b the Mantel-Cox test was used.

In each curve, the smallest step down represents death (or inability to complete the motor task) of one animal in the respective treatment group, bigger steps represent events of multiple animals on the same day.

## Results

### Longevity experiments, grid test and stereology counting

To test whether the applied compounds were effective *in vivo*, HtrA2 KO mice were fed with Idebenone or Resveratrol daily. Both compounds increased life expectancy of HtrA2 KO mice (n = 77 (untreated), n = 25 (Idebenone), n = 34 (Resveratrol); p = 0.0013). Idebenone increased median survival by 7 days (from 32 to 39 days) and Resveratrol by 10 days (to 42 days) (Fig. 1a).

Both compounds significantly delayed onset and progression of disease in HtrA2 KO mice, which usually develop first symptoms consisting of neuromuscular abnormalities around post-natal day 23 leading to death around day 28 [5]. Disease progression was systematically tested by an inverted grid test. Both compounds significantly extend the period of life during which the animals were able to successfully perform the motor task. (Fig. 1b) (n = 5 (untreated), n = 10 (Idebenone), n = 6 (Resveratrol). Untreated animals had a median lifetime of 27 days during which they were able to perform the task. Resveratrol increased this period by 5.5 days to 32.5 days (p = 0.003) and Idebenone increased it by 8.5 days to 35.5 days (p = 0.0001). Both compounds also substantially increased maximal lifetime by which individuals could perform the task. Untreated animals were able to hang onto the grid for at least 30 seconds up to an age of at most 29 days, whereas in the treated groups 10%, respectively 17% of the animals could perform up to an age of at least 48 days for Resveratrol and Idebenone. The maximum lifetime during which the task could be completed was 66 days for Resveratrol and 63 days for Idebenone.

Using a grip strength meter, we attempted to analyze whether the failure to complete the motor task was due to lack of motor strength or loss of coordination (data not shown). However, it was unclear whether the mice released the grip because of lack of coordination or motor strength or both. We could therefore not clearly test whether the KO lead to loss of coordination or strength, or both; similarly we cannot conclude which one of these attributes was improved by Idebenone and Resveratrol.

**Figure 2:**
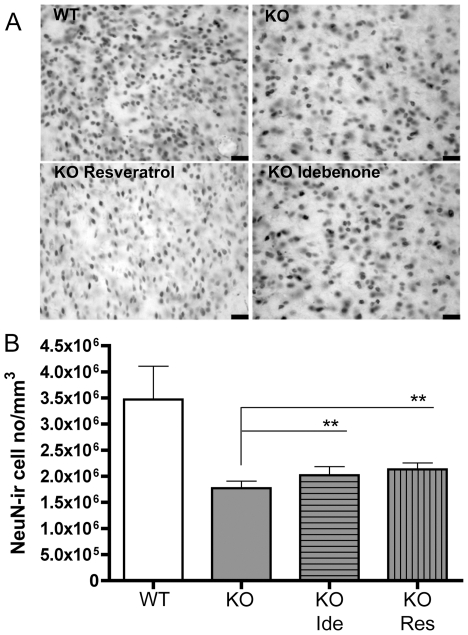
Quantification of neuronal density in dorsal part of striatum. **A** NeuN staining in the dorsal part of the striatum. Scale bar: 280 µm. **B** The treatment with Idebenone and Reseveratrol could significantly attenuate neuronal loss in the dorsal part of the striatum of KO mice. Six mice/treatment group were analyzed.

In order to test if this attenuation of phenotype by Idebenone and Resveratrol is reflected by decreased degeneration of striatal neurons, which had been described as a pathological hallmark in HtrA2 KO mice [5], we examined brains of treated and untreated wt and KO animals sacrificed on post-natal day 30. Lack of the HtrA2 protein resulted in a loss of neurons in the dorsal part of the striatum (p = 0.0001; Fig. 2). Daily supply with either Resveratrol or Idebenone indeed revealed a small but significant increase in neuronal density (p = 0.009 for Idebenone and p = 0.0013 for Resveratrol) when compared to untreated KO animals (Fig. 2). The absolute size of the striatum was constant in all treatment groups. Therefore, these data indicate a rescue effect of total neuron numbers by both compounds.

### CHOP expression in the striatum from HtrA2-KO animals treated with Idebenone and Resveratrol

**Figure 3:**
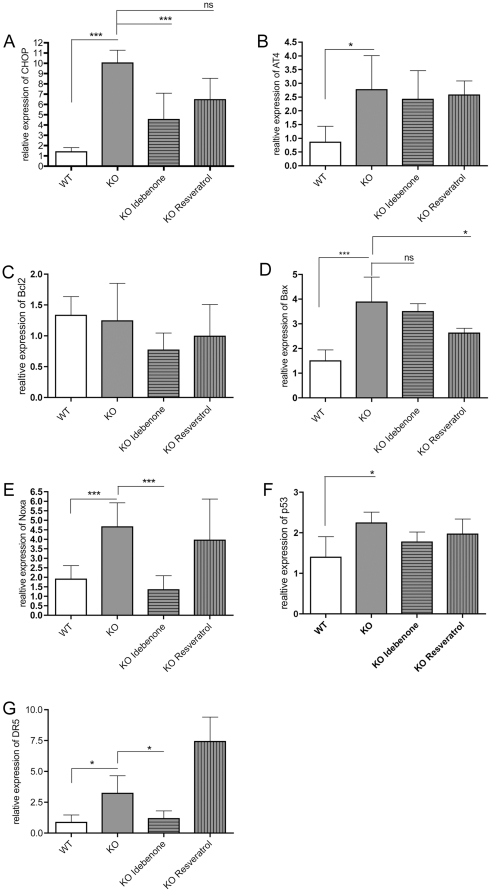
Relative expression of different genes involved in the pathogenesis of HtrA2 KO. **A** Relative expression of CHOP in the striatum. HtrA2 KO animals showed elevated levels of CHOP levels compared to wt. After treatment with Idebenone HtrA2 KO animals showed reduced levels of CHOP. **B** Relative expression of ATF4 in the striatum. Levels of ATF4 were elevated in HtrA2 KO mice compared to wt, but not influenced by Idebenone or Reseveratrol treatment. **C** Relative expression of Bcl2 in the striatum. The relative expression of Bcl2 was not up-regulated in HtrA2 KO animals. Neither treatment with Idebenone nor with Reseveratrol changed the relative expression of Bcl_2_. **D** Relative expression of Bax in the striatum. HtrA2 KO animals exhibited elevated expression levels of Bax. Treatment with Reseveratrol attenuated the up-regulation of Bax. **E** Relative expression of Noxa in the striatum. HtrA2 KO animals showed increased relative expression of Noxa. Treatment with Idebenone of HtrA2 KO animals induced down-regulation of Noxa. **F** Relative expression of p53 in the striatum. HtrA2 KO animals showed upregulated expression levels of p53, while treatment with Idebenone and Reseveratrol had no effect on this. **G** Relative expression of DR5 in the striatum. The elevated levels of DR5 could be reduced after treatment with Idebenone. Empty bars: wt, grey bars: HtrA2 KO. Data are mean± SD; *** p<0.001, ** p<0.01, * p<0.05, ns: not significant; n = 6 mice/group.

To test whether neurodegeneration involves the same pathways described previously [6], we analyzed CHOP mRNA levels in brain tissue taken from striatum of the brain derived from HtrA2 KO mice on day 30 after birth, since this brain area is primarily affected histopathologically [5]. HtrA2 KO mice showed strongly elevated CHOP levels measured in the striatum in comparison to wt mice (Fig. 3a). The degree of CHOP induction was significantly reduced by Idebenone in this brain area to roughly 50% of untreated KO mice. Resveratrol caused a lesser, not significant reduction in levels of CHOP-mRNA (Fig. 3a). Treatment of cerebellar granule neurons of HtrA2 KO mice (see SI Methods) with idebenone prevented the increase of CHOP-mRNA *in vitro* (Fig. S1).

### Examination of signaling cascades involving CHOP in HtrA2 KO mice

Existing data show that induction of CHOP is mainly linked to ER stress inducing the Unfolded Protein Response (UPR) [20] via the up-regulation of transcription factor ATF4, but it was also described as being part of the ISR [21], [6].

Relative expression of the transcription factor ATF4, the possible activator of CHOP, was increased in the striatum of HtrA2 KO mice, whereas treatment with Idebenone or Resveratrol had no effect on its expression (Fig. 3b).

To further address if HtrA2 KO mice displayed different expression patterns of mRNAs linked to the Bcl_2_-family, we analyzed material from the striatum and focused on pro- and anti-apoptotic members, e.g. Bax and Bcl_2_.

HtrA2 KO animals showed no down-regulation for mRNAs of the anti-apoptotic Bcl_2_ gene (Fig. 3c), whereas up-regulation of the pro-apoptotic factor Bax occurred in the striatum (Fig. 3d). Life extension therapy with Resveratrol could decrease the elevated expression of Bax, whereas treatment with Idebenone showed non-significant effect (Fig. 3d).

As shown in Fig. 3e, elevated striatal levels of another pro-apoptotic member of Bcl_2_-family, Noxa, in HtrA2 KO mice declined to wt levels under Idebenone treatment while Resveratrol had no effect. Initial observations indicated that Noxa mRNA is primarily a p53-response gene. To clarify whether up-regulation of Noxa in the brain was dependent on p53, we performed analysis of p53 transcript levels of different brain areas derived from HtrA2 KO animals. Neither Idebenone nor Resveratrol treatment changed the elevated p53 expression levels in the striatum of HtrA2 KO mice significantly (Fig. 3f).

Since CHOP is known to regulate Death Receptor 5 (DR5) expression via binding to its promoter region [22], [23], [24], we next examined if the lack of HtrA2 mediates the activation of DR5 in the striatum of HtrA2 KO mice. This was indeed the case. Treatment with Idebenone reduced elevated expression levels of DR5 in the striatum down to wt levels while Resveratrol had no reducing effect (Fig. 3g).

## Discussion

Despite the controversy on whether HtrA2 might constitute a susceptibility to PD, HtrA2 knockout mice suffer from loss of a certain neuronal populations in the striatum, which resembles a neurodegenerative disorder with a parkinsonian phenotype. Loss of function analysis showed, that HtrA2 has important function in the maintenance of mitochondrial integrity, because absence of the protein leads to accumulation of unfolded proteins in the mitochondria, leading to increased levels of the transcription factor CHOP and cell death, supporting the importance of HtrA2 in the cellular quality control of proteins in neurodegenerative processes [5], [6].

Moreover HtrA2 is sought to regulate constitutive autophagy, which represents an alternative cell death pathway. Therefore HtrA2 induced autophagy by digestion of Hax1 (member of the Bcl2-family), which binds to Beclin-1 (initiator of autophagy) [25]. However, we could not detect differences in the Hax1-expression in wt and HtrA2 KO mice (data not shown).

In the present study we show that Idebenone and Resveratrol have beneficial effects in HtrA2 KO mice *in vivo* and *in vitro*.

Since HtrA2 KO mice are not fertile, progeny have to be bred from heterozygous parents. Therefore only 25% of progeny are HtrA2 KO animals. For *in vitro* experiments we needed to test both compounds in neuronal cell cultures and therefore we resorted to a model of primary neuronal culture that can be prepared from postnatal progeny allowing enough time for genotyping and identification of KO individuals.

Although cerebellar granule neurons are not primarily affected in the brain of HtrA2 KO mice, we provide clear evidence for neurodegeneration in these neurons as indicated by increased CHOP levels [6].

Untreated cultures derived from HtrA2 KO animals showed approximately five fold elevated CHOP levels compared to their wt counterparts. Neither Idebenone nor Resveratrol affected CHOP levels in wt cells, but in HtrA2 KO cultures Idebenone could reduce the amount of CHOP-mRNA to wt levels. Resveratrol had no effect on CHOP expression (Fig. S1; [41]).

**Figure 4:**
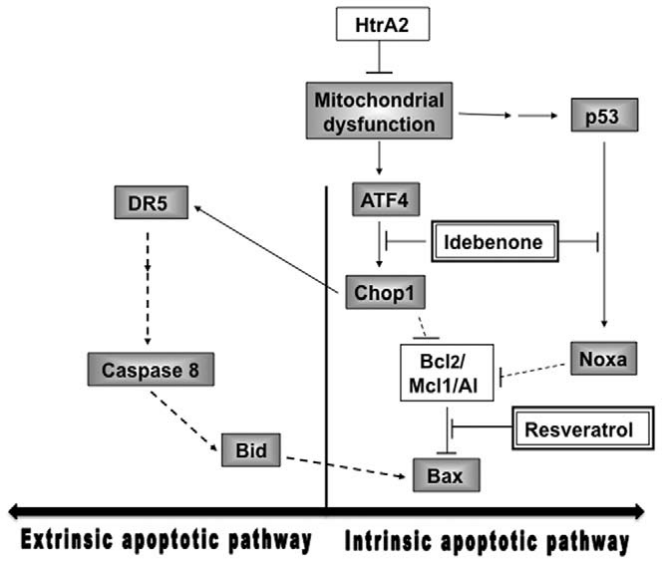
Signaling cascades involving CHOP in the striatum of HtrA2 KO mice. The existence of HtrA2 appears to be crucial for disease progression characterized by mitochondrial dysfunction, which contributes to neuronal cell death via up-regulation of the transcription factors ATF4 and CHOP. CHOP is known to repress Bcl_2_ (pro-survival) gene expression, which increases the proportion of pro-apoptotic Bcl_2_ proteins such as Bax. Oxidative stress also leads to an up-regulation of the pro-apoptotic Bcl_2_- protein Noxa, which can be activated by the transcription factor p53. Noxa exerts its pro-apoptotic function by neutralizing the pro-survival Bcl_2_ protein Mcl1/Al, thereby facilitating the activation of Bax/Bak proteins. CHOP is also known to regulate the extrinstic apoptotic signaling pathway by activating Death Receptor 5 (DR5), a member of the TNFR superfamily, by binding to its promoter region. Idebenone excerts anti-apoptotic effects on CHOP- and Noxa activation downstream of ATF4 and p53, respectively. Idebenone induces also anti-apoptotic effects on DR5 expression. Reseveratrol has been shown to reduce the expression of Bax, downstream of the CHOP- and Noxa activation cascades. Pro-apoptotic factors placed in grey boxes and anti-apoptotic factors placed in white boxes. Antioxidant compounds, used in this study, placed in white boxes with double lines, dashed lines stay for dependent interactions known from literature, solid lines represent dependent interactions drawing from experimental data shown her.

Our experiments show also that although Idebenone and Resveratrol act via distinct pathways, both treatments reduce activation of pro-apoptotic factors. In addition, we were able to identify different signaling steps activated by Idebenone and Resveratrol. A possible model for the molecular way of action of our test compounds is presented in Fig. 4.

HtrA2 appears to be crucial for the maintenance of normal mitochondrial function. Absence of HtrA2 induces disease progression characterized by elevated oxidative stress, followed by up-regulation of the transcription factor CHOP via the activation of ISR and finally apoptosis [6].

Interestingly, both tested compounds extended lifespan, delayed disease onset and increased neuronal density in the striata of treated HtrA2 KO mice. However, Idebenone reduced CHOP expression *in vitro* and *in vivo*, whereas Resveratrol did not. To explain these controversial results, we analyzed the expression of genes from different molecular pathways. There are two major apoptotic signaling pathways: the intrinsic, mitochondria-mediated pathway linked to members of the Bcl_2_-familiy and the extrinsic, death receptor-induced pathway [26]; [27]. CHOP is part of the intrinsic apoptotic pathway and can be activated by the upstream transcription factor ATF4 [28]. Up-regulation of ATF4 represents one upstream element of CHOP activation, and therefore a receptive step in the ISR for life-extending therapy with Idebenone or Resveratrol. Idebenone clearly reduced CHOP levels, but did not influence the levels of expression of the upstream activator ATF4. Idebenone is an orally bioavailable Q10 derivative [9], used for the treatment of Friedreich's ataxia [29]. The strong anti-oxidant properties of Idebenone could explain why treatment promotes down-regulation of the oxidative stress factor CHOP.

Activation of CHOP leads also to up-regulation of diverse downstream targets, which are not yet fully defined, but largely known as initiators of apoptosis. In this regard, CHOP is believed to shift mRNA expression levels of Bcl_2_-family proteins towards pro-apoptotic members, e.g. Bax [21]. Interestingly, HtrA2 KO animals showed no reduced levels of Bcl_2_, but elevated levels of Bax when compared to wt animals. Treatment of HtrA2 KO with Idebenone or with Resveratrol had no significant effect on Bcl_2_-expression. However, we observed a substantial down-regulation of Bax expression after Resveratrol treatment, an effect that might be independent from CHOP. It is more likely that Bax can be activated by other pro-apoptotic factors, linked to the intrinsic and/or extrinsic apoptotic pathway, for example by the BH3 only protein Noxa, which can neutralize the pro-survival Bcl_2_-family protein Mcl1/Al, thereby facilitating activation of Bax/Bak proteins. Interestingly, expression of Noxa was suppressed by Idebenone treatment, while the upstream activator of Noxa, p53 [30], was not affected. Although Idebenone and Resveratrol extended lifespan of HtrA2 KO mice, these compounds did not alter the expression of the upstream elements ATF4 and p53 within the activation cascade of Bax. However, we cannot rule out the possibility that Idebenone and Resveratrol might regulate these transcription factors at translational or post-translational levels.

The intrinsic apoptotic pathway might be linked to the extrinsic pathway, for example through activation of caspase-8 or the bifunctional apoptosis regulator protein BAR [31], leading to an increased expression profile of pro-apoptotic members of the Bcl_2_- family, e.g. BID and Bax/Bak proteins. This amplifying effect triggers the damage of mitochondria, leading to caspase activation downstream of mitochondria [32]. Thus, we examined Death Receptor 5 (DR5), which belongs to the to the Tumor Necrosis Factor Receptor (TNFR) superfamily and is uniquely characterized by the presence of a “death domain“ motif in their cytoplasmic C-terminal region that is crucial for transmitting apoptotic or other signals [33]. Indeed, HtrA2 KO animals showed elevated levels of DR5 mRNA in the striatum, and Idebenone reduced its expression.

Since we did not observe modulation of Bax levels under Idebenone treatment, the regulation of Noxa and DR5 probably alters mitochondrial translocation and oligomerization of Bax [34]; [32], thereby inhibiting apoptosis in HtrA2 KO mice. In contrast, Resveratrol reduced Bax expression, but failed to decrease the expression of other tested genes indicating that these compounds act differentially at the molecular levels.

Resveratrol has been shown to be neuroprotective in *in vivo* and *in vitro* models of PD [35]; [36]. Resveratrol is orally bioavailable but metabolized quickly [37]. However, the effective metabolite is still not known. Resveratrol is one of the active ingredients in grape extract that extended lifespan and improved motor function in a drosophila model of PD due to its antioxidant effect [38]. One of its best studied cell biological targets is the activation of sirtuin 1, a protein involved in aging [39]; [40].

Overall, our results support the feasibility of potential treatment of neurodegenerative diseases like PD with the agents studied here, especially since standard treatment strategies are essentially symptomatic. Since both compounds are known to be well tolerated [29]; [37] and have been administered in patients already, we propose human trials with Resveratrol and Idebenone especially in patients carrying the HtrA2/Omi mutation.

In summary, our data suggests that Idebenone acts upstream on the expression levels of CHOP but downstream of ATF4, and upstream of Noxa but downstream of p53 in the intrinsic apoptotic pathway (Fig. 4). Regarding the extrinsic pathway Idebenone appears to exert pro-survival effects on DR5 expression via CHOP down-regulation. Conversely, Resveratrol seems to act only as Bax regulator in this context. In any case, both reagents induce a decreased activation of pro-apoptotic factors and thereby extend the survival rate of HtrA2 KO mice.

## Supporting Information

Figure S1 (TIF)

Methods S1 (DOCX)
