# Supplementary figures and images for: Idebenone and Resveratrol Extend Lifespan and Improve Motor Function of HtrA2 Knockout Mice
Source: PLoS One. 2011 Dec 19;6(12):e28855. doi: 10.1371/journal.pone.0028855 (PMC3242749; doi:10.1371/journal.pone.0028855)

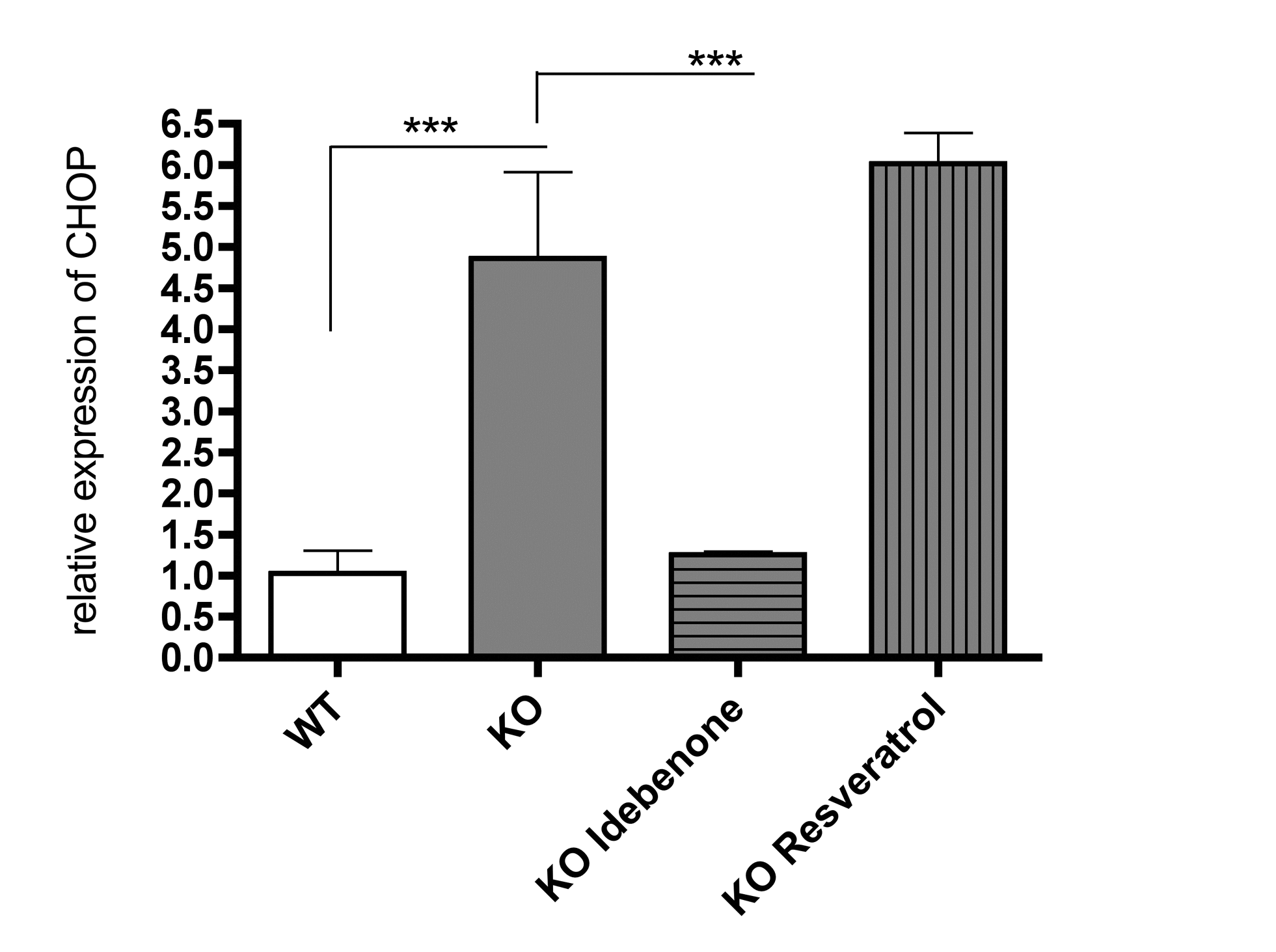

Supplement: Figure S1 — (TIF) [file pone.0028855.s001.tif]
