# Supplementary material for: Idebenone and Resveratrol Extend Lifespan and Improve Motor Function of HtrA2 Knockout Mice
Source: PLoS One. 2011 Dec 19;6(12):e28855. doi: 10.1371/journal.pone.0028855 (PMC3242749; doi:10.1371/journal.pone.0028855)
Supplement: Methods S1 — (DOCX) [file pone.0028855.s002.docx]

**Supplemental methods and data**

**Cerebellar granule neurons (CGCs)** were prepared from 7-day-old WT and HtrA2 KO mice [5] as described previously [41]. Cells were dissociated in the presence of trypsin and DNaseI, and plated in dishes coated with poly-L-lysine (10 mg/ml). Granule cells were seeded at a density of 3x10^5^ cells/cm^2^ in basal modified Eagle’s medium (Biochrom AG, Berlin, Germany) supplemented with 10% FBS (Gibco, Invitrogen, Darmstadt, Germany), gentamicin (20 mg/mL), and L-glutamine (2 mmol/l). Cells maintained in depolarizing conditions were supplemented with 20 mmol/l KCl to achieve a final concentration of 25 mmol/l K+. Cytosine1-ß-D-Arabinofuranoide (10 mmol/l, Sigma, Taufkirchern, Germany) was added to the culture medium after 24h to arrest the growth of non-neuronal cells. Neurons were also cultured 24 h after plating in BME-media containing Resveratrol (25 μM, Tocris, Ellisville, Ms, USA) or Idebenone (4 μM, Santhera, Pharmaceuticals Holding AG, Liestal, Schweiz). Cultures generated by this method have been characterized and shown to contain >95% granule neurons [41]. After 7 days in culture, total RNA has been isolated, and c-DNA synthesis was performed according to the company’s instructions (iScript, Bio-Rad, München, Germany). For each treatment/ four wells (six pictures/well) have been analyzed. The experiment has been repeated for three times.
